# Are Numbers, Size and Brightness Equally Efficient in Orienting Visual Attention? Evidence from an Eye-Tracking Study

**DOI:** 10.1371/journal.pone.0099499

**Published:** 2014-06-16

**Authors:** Hermann Bulf, Viola Macchi Cassia, Maria Dolores de Hevia

**Affiliations:** 1 Department of Psychology, University of Milano-Bicocca, Milano, Italy; 2 Université Paris-Descartes, Laboratoire Psychologie de la Perception, CNRS, UMR 8242, Paris, France; 3 Cognitive Neuroimaging Unit, NeuroSpin, INSERM, U992, Gif sur Yvette, France; University of Melbourne, Australia

## Abstract

A number of studies have shown strong relations between numbers and oriented spatial codes. For example, perceiving numbers causes spatial shifts of attention depending upon numbers' magnitude, in a way suggestive of a spatially oriented, mental representation of numbers. Here, we investigated whether this phenomenon extends to non-symbolic numbers, as well as to the processing of the continuous dimensions of size and brightness, exploring whether different quantitative dimensions are equally mapped onto space. After a numerical (symbolic Arabic digits or non-symbolic arrays of dots; Experiment 1) or a non-numerical cue (shapes of different size or brightness level; Experiment 2) was presented, participants' saccadic response to a target that could appear either on the left or the right side of the screen was registered using an automated eye-tracker system. Experiment 1 showed that, both in the case of Arabic digits and dot arrays, right targets were detected faster when preceded by large numbers, and left targets were detected faster when preceded by small numbers. Participants in Experiment 2 were faster at detecting right targets when cued by large-sized shapes and left targets when cued by small-sized shapes, whereas brightness cues did not modulate the detection of peripheral targets. These findings indicate that looking at a symbolic or a non-symbolic number induces attentional shifts to a peripheral region of space that is congruent with the numbers' relative position on a mental number line, and that a similar shift in visual attention is induced by looking at shapes of different size. More specifically, results suggest that, while the dimensions of number and size spontaneously map onto an oriented space, the dimension of brightness seems to be independent at a certain level of magnitude elaboration from the dimensions of spatial extent and number, indicating that not all continuous dimensions are equally mapped onto space.

## Introduction

Numbers and space appear to be intimately related in the human mind. Current models of numerical processing postulate the existence of an analogue-quantity code where numbers are represented as variable distributions of activation over an oriented *mental number line* [1], [2]. One of the most convincing evidence of this number-space mapping is the Spatial Numerical Association of Response Codes (SNARC) effect [3]: In numerical classification tasks, relatively large numbers are responded to faster with a right response than with a left response, whereas relatively small numbers are responded to faster with a left response than with a right response. The SNARC effect is usually interpreted in terms of an internal representation of numbers which is organized on a horizontal number line, with small numbers on the left side and large numbers on the right side, at least in Western cultures [3]–[5]. Moreover, the finding that the SNARC effect is present even when numerical information is irrelevant to the task [4], [6] is taken as evidence for a relatively automatic access to the spatial components of numerical information.

Spatially-related effects consistent with a spontaneous mapping of number onto an oriented space emerge also in other types of behavioral tasks involving numerical processing, such as stimulus detection [7], line bisection [8], and in neuropsychological studies with neglect patients, who are unable to attend to the contralesional side of space (i.e., the left side) and exhibit deficits in numerical tasks that tap onto an oriented spatial representation of number [9], [10]. Recent comparative and developmental studies offer evidence that even non-human animals [11] and preverbal human infants [12] establish a spontaneous relationship between numerical ordering and a left-to-right oriented axis. At the age of four, preschool children exhibit spontaneous intuitions about the spatial dislocation of numbers in different spatial-numerical tasks [13] and, eventually, an oriented number-space mapping is firmly established by the school years [14], [15].

A further line of evidence supporting the argument of a spatial mapping of numbers along a left-to-right oriented axis is that numerical information modulates performance in visuo-spatial tasks [16], [17]. For example, Fischer et al. [17] showed that merely looking at Arabic digits causes a shift in attention to either the right or left visual field depending on the digit's numerical magnitude. In particular, targets presented on the right side of the screen are detected faster after a large number (i.e., 8 or 9) is presented centered on the screen than after a small number (i.e., 1 or 2), and vice versa for targets presented on the left side of the screen. Based on these findings, the authors concluded that perceiving numbers influences the allocation of spatial attention within the visual field. In the same vein, it has been shown that numbers can influence saccadic eye movements, whereby subjects exhibit faster gaze responses towards the left visual hemi-field when they categorize small numbers and towards the right visual hemi-field when they categorize large numbers [18]. These findings fit well with evidence from neuroimaging studies showing that neural circuits dedicated to eye movements are recruited during arithmetic, suggesting that mental arithmetic co-opts parietal circuitry associated with spatial coding [19].

Overall, these findings are in line with the ATOM (A Theory Of Magnitude) model proposed by Walsh [20], according to which numbers might not be represented in isolation but spontaneously connected to space representation. Importantly, the ATOM model also posits that such connection with space representation might not be unique to number, since all ordered magnitudes would be represented in the brain according to a common metric that is inherently spatial in nature. In the current study we investigated whether non-numerical magnitude information can drive the allocation of visual attention in the visual field in the same way as numbers do. Specifically, we presented adults with both symbolic and non-symbolic numerical notations -i.e., Arabic digits and sets of dots - and non-numerical continuous quantities -i.e., size and levels of brightness. As for numerical quantities, neuroimaging studies offer evidence for the existence of a common neural code underlying the representation of both symbolic and nonsymbolic number [21], [22]. Moreover, there is behavioral evidence that nonsymbolic numbers are mapped into space in the same way as symbolic numbers, as shown by studies testing illiterate adults living in an Amazonian remote culture, with little or no education [23], preschool-aged children prior to exposure to formal education [24], and preverbal infants [25], [26]. On this ground, we hypothesized that both Arabic digits and dot arrays could act as a meaningful cue in driving visual attention to either the right or left visual field depending on their magnitude.

Most crucially, our attempt to verify whether the continuous dimensions of size and/or brightness could exert the same effect as number on the allocation of visual attention was motivated by inconsistent evidence suggesting that the number-space mapping extends to magnitudes which have no numerical value. On the one hand, a SNARC effect has been described for the dimensions of physical size, brightness and conceptual size [27], and mutual interference effects at both the behavioral and neural level have been described within a Stroop-like paradigm for the dimensions of number, size and brightness [28]. On the other hand, it has been shown that, among various possible mappings between dimensions, the number-space mapping might have a privileged status [29]–[32]. For example, recent developmental research showed that when preschool children are tested for their ability to create cross-dimensional matches between different instances from the dimensions of number, line length, and level of brightness, they reliably perform mappings between number and length, whereas they are only partially capable to match brightness and length, and fail to match brightness and number [29]. Moreover, 8-month-old infants can spontaneously transfer the discrimination of an ordered series of numerosities to the discrimination of an ordered series of line lengths [25] but create less reliable relationships between numbers and levels of brightness [30], suggesting that even in preverbal stages of human development the number-space mapping might have a special status. It is therefore an open question whether human adults map all continuous magnitudes onto the spatial dimension in the same way as they do for numbers, and therefore whether any visuo-spatial phenomena described for numbers extend as well to any continuous dimension.

In the present study we measured the latency of participants' eye movements within a cued visual detection task in order to investigate whether the lateralized shifts of attention induced in adults by Arabic digits [17] are observed when magnitude information is provided by non-symbolic number, and/or by non-numerical continuous dimensions such as physical size and brightness. A visual target was presented either on the left or the right side of a screen after the onset of a small-magnitude or a large-magnitude cue that appeared in the center of the screen. In Experiment 1 the cue was a symbolic (i.e., an Arabic digit) or a non-symbolic (i.e., a set of dots) numerical stimulus, whereas in Experiment 2 the cue was a shape that varied either in physical size or in brightness. Using an automated eye-tracking system, we measured the time to target fixation under free looking condition. In order to eventually draw parallelisms with infants' studies, and based on evidence of spontaneous attentional and visual behavior related to numbers [19], [33], [34], adults were not given any explicit instructions, and therefore their spontaneous behavior was measured. We predicted that, if any magnitude cue is spontaneously mapped onto space, participants would be faster at detecting (i.e., orienting towards) targets presented on the right side of the screen after the presentation of a large-magnitude cue, relative to a small-magnitude cue, and the reverse for targets presented on the left side of the screen.

## Experiment 1

Experiment 1 investigated whether small-magnitude (i.e., 2) and large-magnitude (i.e., 9) Arabic digits and arrays of dots induce spontaneous shifts of visual attention, by modulating the time required to fixate the target that appeared either on the left or the right side of the visual field.

### Method

#### Ethic Statement

The protocol was carried out in accordance with the ethical standards of the Declaration of Helsinki (BMJ 1991; 302: 1194) and approved by the Ethics Committee of the University of Milano Bicocca. All participants signed an informed consent before testing.

#### Participants

The sample included 16 adults (12 females; age: mean 24,44 years, range 20–42). All participants had normal or corrected-to-normal vision. They had no previous experience of eye-movement studies and were naive to the hypotheses of the experiment.

#### Stimuli, apparatus and procedure

Participants were placed 60 cm from the stimulus monitor. Before beginning the experimental trials, the eye tracker was calibrated by presenting animated cartoons at nine different locations on the stimulus monitor. Subsequent eye movement data were calculated from these calibration values.

**Figure 1:**
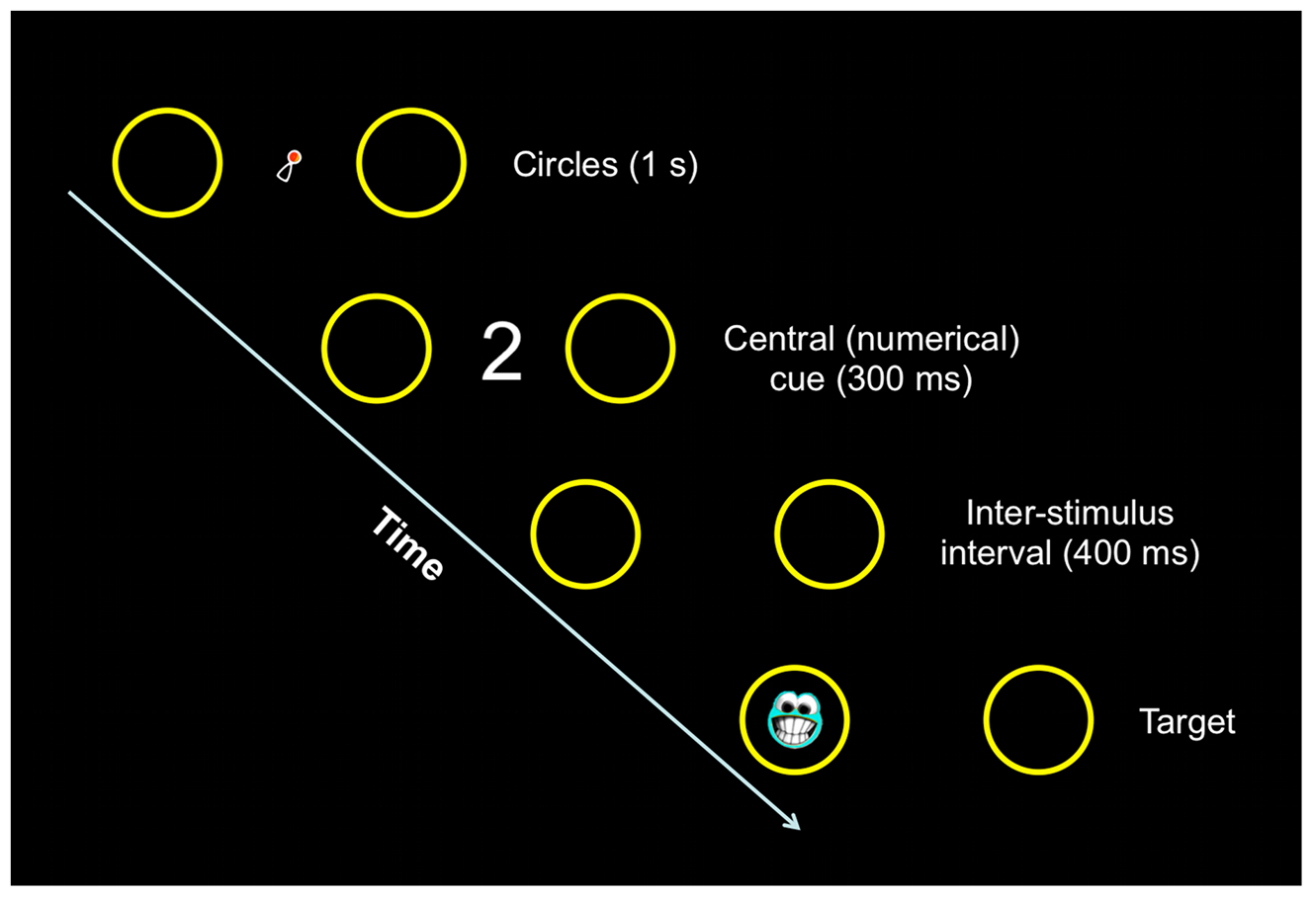
Schematic representation of a congruent trial in the Arabic digits condition of Experiment 1.

**Figure 2:**
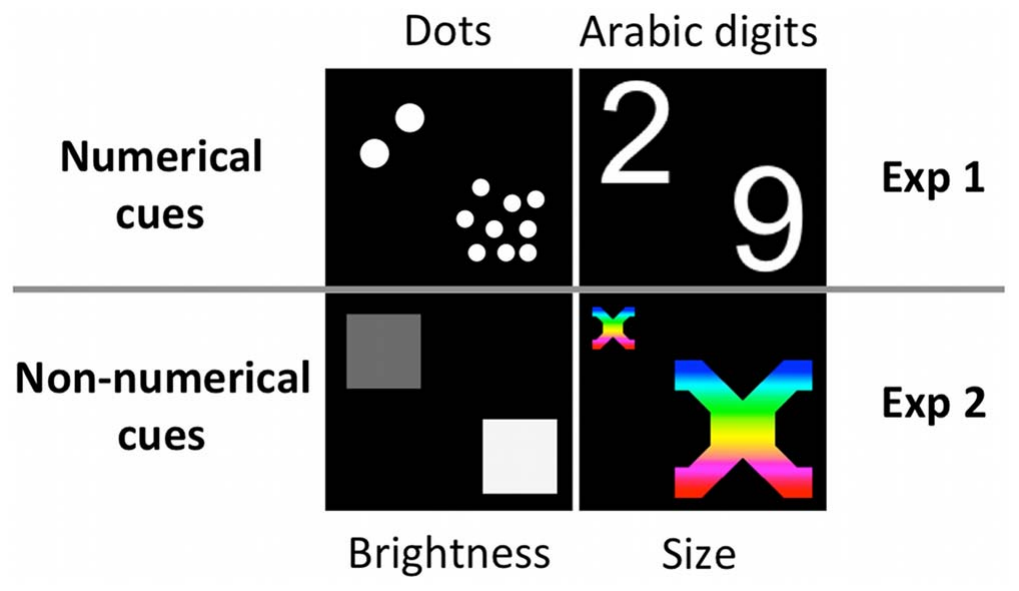
Numerical and non-numerical magnitudes presented as cues in Experiment 1 and in Experiment 2, respectively.

Each experimental trial began with the presentation, in the center of the screen, of an attention getter (animated cartoon) appearing on a black background. As soon as the participant looked at the attention getter for 300 ms, two colored circles (6°) were automatically presented peripherally (11° of eccentricity, with the two edges of the circles separated by 16°), one at the left and one at the right side of the central attention getter (Figure 1). Circles of three different colors (red, yellow and blue), and three different types of attention getters were randomly presented across the experimental trials. The central attention getter remained present on the screen until a cue appeared in the center of the screen, which occurred 1000 ms after the appearance of the circles. The cue remained on the screen for 300 ms and, after an Inter-Stimulus Interval (ISI) of 400 ms, a target consisting of a flickering schematic face (3.2°) appeared within one of the two peripheral circles. The target remained visible until the participant looked at it or for a maximum of 2 s. This terminated the trial, and another trial began with the appearance of the central attention getter. On each trial, one out of three different target types was randomly presented within either the left or the right circle. The cue consisted of a small magnitude (i.e., ‘2’) or a large magnitude (i.e., ‘9’) number appearing either in symbolic (i.e., Arabic digits) or in non-symbolic notation (i.e., array of dots) (Figure 2). Arabic numbers, as well as the 2-dot and the 9-dot arrays, were controlled for overall area. Arabic numbers were 1.9° by 2.8° of visual angle, and the virtual square occupied by the dot arrays was 3.5° by 3.5°. Two 2-dot arrays (one oriented leftwards, and one oriented rightwards), and four 9-dot arrays were used.

The experiment consisted of two 60-trial blocks, the first one in which non-symbolic numerical stimuli were presented, the second one in which symbolic numerical stimuli were presented. Each block consisted of 48 experimental trials (2 numerical display ×2 target position ×12 repetitions), and 12 catch trials. Catch trials were introduced to prevent anticipatory responses, and did not include the target. The magnitude (2 or 9) of the numerical display and the position (left or right) of the target were randomized across trials. The stimuli were presented with E-Prime 2.0 software on a 24″ monitor with a resolution of 1600×1200 pixels, and eye movements were recorded using an ASL6 remote eye-tracking system at a frequency of 120 Hz (Applied Science Laboratory, MA). To coordinate the eye movement data with the respective stimulus displays, the stimulus-generating computer sent unique, time-stamped numerical codes via a parallel port to the ASL computer, indicating the onset and type of stimulus display. In turn, the ASL computer sent the coordinates of the eye-movements continuously to the stimulus-generating computer that computed the coordinates of the eye-movements using E-Prime 2.0.

#### Data analysis

The visual display was virtually divided into 3 areas of interest (AOI), one surrounding the position of the central attention getter, and the other two corresponding to the two peripheral circles where the targets appeared. Each AOI measured approximately 12.6° in width, and 7.6° in height. Time to target fixation (TTF) was used as the dependent variable. TTF refers to the time difference between the target onset and the time the participant's gaze entered the target AOI, provided that the AOI was fixated for at least 100 ms.

Analyses were performed on the 96 experimental trials. Catch trials were not included in the analyses. Experimental trials were divided into congruent and incongruent trials, with reference to the orientation of the mental number line. On congruent trials a small-magnitude cue was followed by a target appearing in the left AOI, and a large-magnitude cue was followed by a target appearing in the right AOI. On incongruent trials a small-magnitude cue preceded the appearance of a target in the right AOI, and a large-magnitude cue preceded the appearance of a target in the left AOI.

### Results

An average of 15.5 trials (standard error of the mean, SEM = 2.22) were excluded from the statistical analyses for the following reasons: (i) the participant did not look at the central AOI at the onset of the peripheral circles, the cue and/or the target; (ii) the participant did not enter the AOI that contained the target; (iii) the signal of the eye tracker was lost during stimulus presentation. The final mean number of trials included in the analyses was 80.5 (SEM  = 2.22) for each participant.

**Figure 3:**
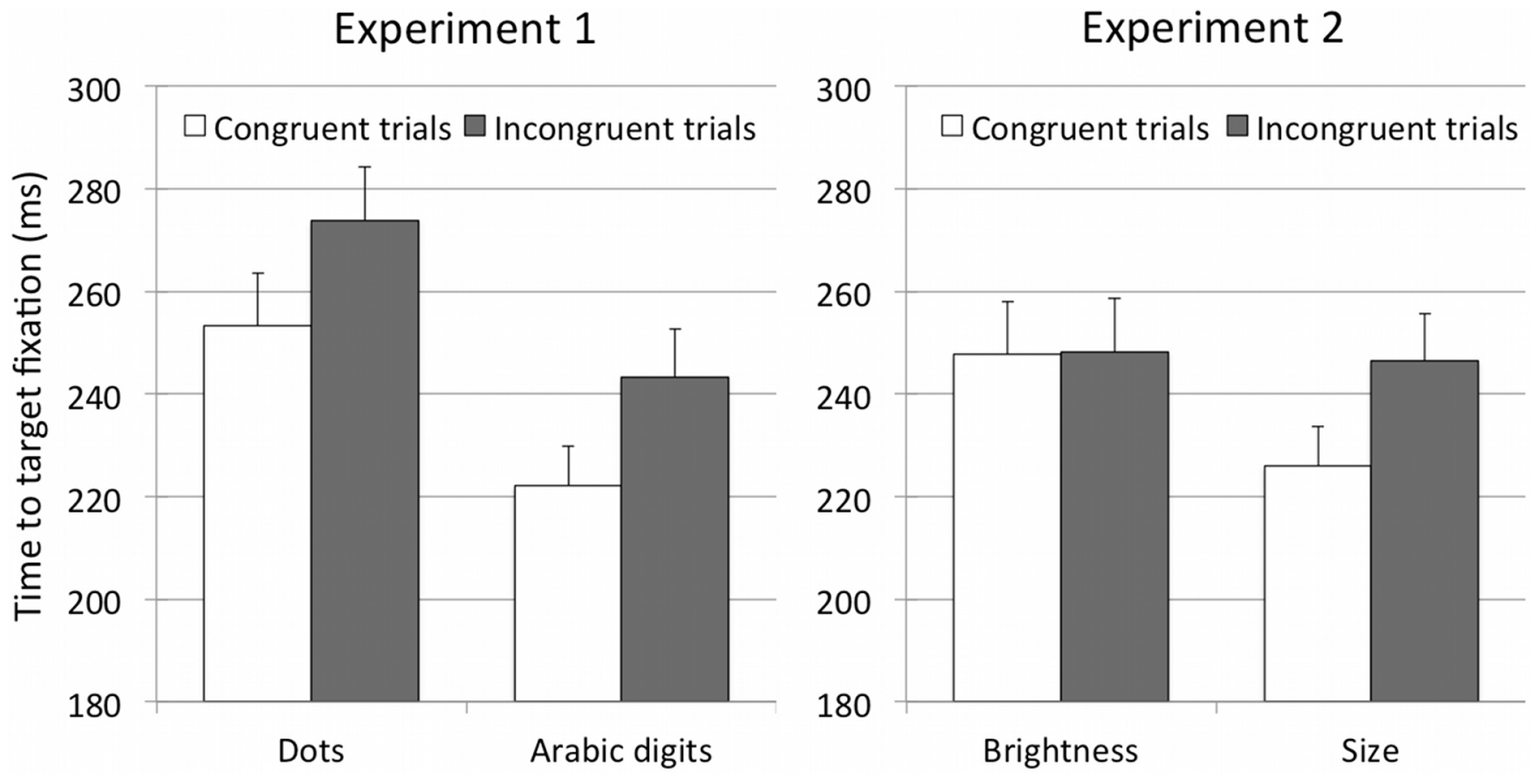
Mean times to target fixation exhibited by participants on congruent and incongruent trials for the Dots and Arabic digits conditions of Experiment 1 and for the Brightness and Size conditions of Experiment 2. Error bars represent standard error of the means.

Time to target fixations (TTFs) were submitted to a repeated-measures ANOVA with Number display (Arabic digits vs. dots) and Cue-Target congruency (congruent vs. incongruent) as within-subjects factors. The analysis yielded a significant main effect of Number display, F(_1,15_) = 9.1, p = 0.009, η_p_^2 = ^0.38, with faster TTFs at detecting the target when it was cued by Arabic numbers (M = 232.65 ms, SEM = 6.22) compared to dot arrays (M = 263.52 ms, SEM = 7.4). Critically, there was also a main effect of congruency, F(_1,15_) = 14.41, p = 0.002, η_p_^2 = ^0.49, with faster TTFs at detecting targets on congruent trials (M = 237.65 ms, SEM = 6.87) compared to incongruent trials (M = 258.53 ms, SEM = 7.39). The interaction between the two factors was not significant, F(_1,15_) = 0.002, p = 0.97, η_p_^2^<0.001, and planned comparisons performed through paired-samples *t-*tests confirmed that TTFs were faster on congruent trials compared to incongruent trials both when the numerical notation of the cue was symbolic (M = 222.07 ms, SEM = 7.65 vs. M = 243.23 ms, SEM = 9.31), t(_15_) = 2.69, p = 0.017, and when the notation of the cue was non symbolic (M = 253.22 ms, SEM = 10.21 vs. M = 273.81 ms, SEM = 10.4), t(_15_) = 2.22, p = 0.043 (Figure 3).

Examination of the data for individual participants through binomial tests confirmed the results of the analysis on TTF, revealing that 12 out of 16 participants (12 vs. 4, p = .077) in the dots condition, and 13 out of 16 participants (13 vs. 3, p = 0.021) in the digits condition showed faster response times to the target in the congruent trials than in the incongruent ones.

### Discussion

Results from Experiment 1 show that targets appearing within the right circle were detected faster when preceded by large numbers and left targets were detected faster when preceded by small numbers, both in the case of Arabic digits and dot arrays. These findings replicate earlier demonstrations that symbolic number can drive the allocation of visuo-spatial attention within the visual field [17], and further extend them to non-symbolic number represented by sets of dots.

The results also show that participants were significantly faster at detecting targets cued by Arabic numbers compared to dot arrays. A similar advantage in adults' performance for symbolic over non-symbolic numerical notations has been previously reported by de Hevia and Spelke [24, Experiment 2], who found that adults were faster at detecting targets that appeared at the location of Arabic numbers vs. dot arrays within a dot-probe task. This finding might stem from the relative visual complexity of the dot arrays relative to the digits. In any event, and more crucial to the aim of the current study, the phenomenon of biased visual attention related to number magnitude was observed for both types of numerical stimuli.

Overall, findings from Experiment 1 provide evidence that the mere perception of a number, in either symbolic or non-symbolic notation, induces attentional shifts to a peripheral region of space that is congruent with the numbers' relative position on a mental number line. The close correspondence between the findings obtained with symbolic digits and those obtained with non-symbolic arrays supports the view of an abstract representation of numerical information [21].

As already discussed, the ATOM model [20], [35] posits that other magnitudes besides number might be spontaneously connected to space representation. In line with this hypothesis, in Experiment 2 we investigated whether the finding that looking at numbers causes a shift of attention to the left or the right side of the visual field may be extended to non-numerical ordered continuous dimensions, such as physical size and brightness.

## Experiment 2

Experiment 2 tested whether non-numerical magnitude information is spontaneously mapped onto an oriented space, and, specifically, whether previous demonstrations of numerical effects on the allocation of visuo-spatial attention extend to other magnitude dimensions. To this end, we tested whether physical size (i.e., small- vs large-sized shape) and brightness level (i.e., low vs high brightness) modulate performance in the same way as numerical information in the same visuo-spatial task used in Experiment 1. We hypothesized that, if any ordered continuous dimension maps onto space as numbers do, and these mappings display the same functional properties as the number-space mapping does, then the same pattern of results found in Experiment 1 should be observed also for the dimensions of size and brightness. Accordingly, time to target fixation to the left and to the right side of the visual field should be modulated by the information of magnitude provided by the cue.

### Method

#### Participants

The final sample included 17 adults (14 females, age: age: mean 24,06 years, range 19–49 years). All participants had normal or corrected-to-normal vision, they had no previous experience of eye-movement studies and were naive to the hypotheses of this experiment.

#### Stimuli, apparatus, procedure and data analysis

Stimuli, apparatus, procedure and data analysis were the same as in Experiment 1, with the exception that non-numerical magnitudes, instead of numerical magnitudes, were presented as central cues before the presentation of the peripheral target (Figure 2). The non-numerical cues consisted of two geometrical shapes varying in their level of brightness according to a 1∶4.5 rgb ratio (i.e., a square and a hexagon; range  = 51 rgb to 229.5 rgb), or two rainbow-colored shapes varying in their physical size according to a 1∶4.5 ratio (i.e., an X-shaped figure and an equilateral cross with four arms bent at 90°; range  = 2.77 cm^2^ to 12.5 cm^2^). The two shapes that varied in brightness were equated in overall amount of area (16 cm^2^) and were presented against a black background, so that the brighter figure had also the higher contrast [see also 29,30,36]. As in Experiment 1, participants were administered two 60-trial blocks. All participants completed the trials in which the cue varied in brightness before being administered the trials in which the cue varied in size.

### Results

A mean of 14.4 trials (SEM = 1.87) were excluded from the statistical analyses for the following reasons: (i) the participant did not look at the central AOI at the onset of the peripheral circles, the cue and/or the target; (ii) the participant did not enter the AOI that contained the target; (iii) the signal of the eye tracker was lost during stimulus presentation. The final mean number of trials included in the analyses was 81.6 (SEM = 1.879) for each participant.

TTFs were analyzed through a repeated-measures ANOVA with Dimension (Brightness vs. Size) and Cue-Target congruency (Congruent vs. Incongruent trials) as within-subjects factors. The analysis revealed marginally significant main effects of Dimension, F_1,16_ = 3.75, p = 0.07, η_p_^2 = ^0.19, and congruency, F_1,16_ = 3.76, p = 0.07, η_p_^2 = ^0.19, and a significant interaction between the two factors, F_1,16_ = 9.28, p = 0.008, η_p_^2^ = 0.37. Planned comparisons performed through paired-samples *t-*tests revealed that TTFs were faster on congruent trials compared to incongruent trials only when the cue varied in physical size (M = 225.95 ms, SEM = 6.96 vs. M = 246.38 ms, SEM = 6.74), t_16_ = 4.15, p = 0.001, but not when the cue varied in brightness (M = 247.66, SEM = 7.53 vs. M = 248.11, SEM = 6.04), t_16_ = 0.06, p = 0.95 (Figure 3).

Examination of the data for individual participants through binomial tests confirmed the results of the analysis on TTF, revealing that 9 out of 17 participants (9 vs. 8, p = 1) in the luminance condition, and 14 out of 17 participants (14 vs. 3, p = 0.013) in the size condition showed faster response times to the target in the congruent trials than in the incongruent ones.

To compare the efficiency of physical size to that of number in orienting visual attention participants' performance in the size condition of Experiment 2 was compared to participants' performance in the number conditions of Experiment 1. Since Arabic digits and dots were tested within subjects, we performed two separate ANOVAs with Cue-Target congruency (congruent vs. incongruent trials) as within-subjects factor and Dimension (number vs. physical size) as between-subjects factor, in which, the size condition was compared to the Arabic numbers and the digits condition respectively. The analyses yielded a main effect of Dimension in the size vs. dots comparison, F_1,31_ = 6.08, p = 0.019, η_p_^2^ = 0.16, with faster TTFs at detecting the target when it was cued by size (M = 236.17 ms, SEM = 5.09) compared to dot arrays (M = 263.52ms, SEM = 7.4). The main effect of Dimension in the size vs. Arabic numbers comparison was not significant F_1,31_ = 0.13, p = 0.72. Moreover, both analyses revealed a significant main effect of congruency (size vs. digits: F_1,31_ = 20.63, p<0.001, η_p_^2^ = 0.40; size vs. dots: F_1,31_ = 15.73, p<0.001, η_p_^2^ = 0.34), with TTFs being faster on congruent trials compared to incongruent trials irrespective of the numerical or non-numerical information embedded in the cue. No interactions were significant (ps>.93).

### Discussion

The results of Experiment 2 show that right targets were detected faster when preceded by large-sized shapes and left targets were detected faster when preceded by small-sized shapes, suggesting that size is another dimension of magnitude that, like number, maps onto an oriented spatial extent and effectively orients visuo-spatial attention. In contrast, cues varying in their level of brightness did not modulate the speed of target detection. These findings fit well with earlier demonstrations of weak cross-dimensional mapping between brightness and other magnitude dimensions in adults [31], [32], children [29], and infants [30]. Together, this evidence suggests that the dimension of brightness is independent at a certain level of magnitude elaboration from the dimensions of spatial extent and number.

The results also show that participants were faster at detecting targets cued by shapes varying in physical size compared to dot arrays. In line with findings from Experiment 1, the visual complexity of the dots arrays compared to a single shape might explain the overall temporal advantage for the size condition compared to the non-symbolic number condition.

## General Discussion

Capitalizing on the finding that Arabic numbers can orient visuo-spatial attention towards the left/right depending on their numerical magnitude [17], the present study investigated whether different sources of magnitude information, besides symbolic number, can drive the orientation of spatial attention in the visual field. After being cued with numerical (i.e., symbolic Arabic digits and non-symbolic arrays of dots, Experiment 1) or non-numerical stimuli (i.e., geometrical shapes that varied in level of brightness or in physical size, Experiment 2), participants were presented with a target that could appear either on the left or the right sides of the screen.

Experiment 1 shows that when targets appeared on the right they were detected faster if preceded by large numerical magnitudes, while when targets appeared on the left they were detected faster when preceded by small numerical magnitudes, both for Arabic digits and dot arrays. These results indicate that looking at a number, irrespective of its symbolic or non-symbolic formats, induces attentional shifts towards a peripheral region of space that is congruent with the numbers' relative position on a mental number line. The finding that also numbers represented by dot arrays produce these effects suggests that the spontaneous tendency to associate numbers to different spatial positions does not directly derive from experience with measurement devices, such as rulers or graphs, where digits are canonically presented from left to right. We believe this finding should encourage researchers to explore whether a predisposition to relate numerical ordering and a left-to-right oriented axis emerges in early stages of development before any formal education has taken place, and while the influence of cultural conventions is still limited. On this view, an experimental paradigm based on the recording of eye-movements in a free-looking condition, like the one we used in the current study, might be useful to investigate whether magnitude information can drive the orienting of visual attention in preverbal infants, who cannot receive verbal instruction and who are poor at motor responses. Indeed, the recording of eye-movements in a free-looking condition has been recently demonstrated to be a suitable tool to assess the functioning of visual attention in few-month-old infants [37], [38].

Results from Experiment 2 show that non-numerical cues modulate the deployment of visuo-spatial attention only in the case of physical size, but not in the case of brightness. Targets appearing on the right side were detected faster when preceded by a large-sized shape and targets appearing on the left were detected faster when preceded by a small-sized shape. However, differences in the level of brightness of a geometrical shape did not modulate the timing of target detection. Although SNARC effects have been reported for other non-numerical dimensions such as brightness, size, and conceptual size [27], this study suggests that not any dimension that is conceptualized in terms of an oriented horizontal line has specific modulating effects in visuo-spatial tasks. The phenomena described here for the dimensions of number and size is in line with the view that not all continuous dimensions are processed in the same way, both at the functional [29], [30], [32] and neural levels [31].

In summary, our results confirm the role of symbolic numerical magnitude in orienting spatial attention [17], and extend this effect to non-symbolic numbers, supporting the view of an abstract representation of numerical representation [21]. Moreover, the finding that some but not all continuous dimensions modulate visuo-spatial attention in the same way as number does, supports the view of a privileged link between the dimensions of number, size and space, that does not extend to other ordered magnitudes, such as brightness. Future research might uncover whether other ordered information that does not include any magnitude dimension, such as days of the week and letters of the alphabet, are able to exert the same modulating effects on visuo-spatial processing [32].
